# Supplementation of γ-Aminobutyric Acid as a Functional Nutrient

**DOI:** 10.3390/nu18142389

**Published:** 2026-07-22

**Authors:** Wei-Yang Lu

**Affiliations:** Department of Physiology and Pharmacology, Schulich School of Medicine and Dentistry, University of Western Ontario, London, ON N6A 5B7, Canada; wlu53@uwo.ca

**Keywords:** γ-aminobutyric acid, functional nutrient, gut microbiota, intestinal absorption, hepatic metabolism, GABA shunt, immune regulation, metabolic regulation

## Abstract

γ-Aminobutyric acid (GABA) is an endogenous metabolite and signaling molecule that is widely distributed across plants, microorganisms, and mammalian tissues. Although classically viewed as the principal inhibitory neurotransmitter in the central nervous system, GABA also functions in peripheral organs, where it participates in receptor-mediated signaling, intermediary metabolism, epithelial barrier regulation, endocrine control, immune modulation, and host–microbiota communication. These features have renewed interest in oral GABA supplementation and in dietary or microbial strategies designed to increase luminal or circulating GABA availability. Despite increasing interest in oral GABA supplementation and microbiota-derived GABA, evidence remains fragmented across multiple disciplines and the translational relevance of peripheral GABA biology remains incompletely defined. This review considers GABA within a functional nutrient framework: not as an essential nutrient required to prevent deficiency, but as a nonessential bioactive metabolite whose exogenous availability may modulate physiological regulation under specific conditions. The literature was identified through structured PubMed searches of studies published from 2000 onward. This review summarizes current knowledge regarding exogenous sources of GABA, intestinal absorption, hepatic uptake, and metabolic fate, receptor-mediated and metabolite-mediated signaling mechanisms in peripheral tissues, and findings from experimental, preclinical, and clinical studies examining GABA supplementation in immune-, endocrine-, and metabolic-related contexts. The findings support GABA as a biologically active functional nutrient with potential roles in immune, endocrine, epithelial, hepatic, and metabolic regulation. While experimental studies consistently report beneficial effects on inflammatory and metabolic outcomes, human studies remain limited and have not yet established definitive clinical efficacy. Future studies should prioritize well-powered randomized controlled trials, dose–response analyses, long-term safety assessments, and biomarkers of tissue-specific GABA exposure and target engagement.

## 1. Introduction

γ-Aminobutyric acid (GABA) is a non-proteinogenic amino acid that is conserved across plants, microorganisms, and tissues of mammals including humans. Although GABA is classically recognized as the principal inhibitory neurotransmitter in the mammalian central nervous system (CNS), increasing evidence indicates that its biology extends far beyond neurotransmission [1]. In peripheral tissues, GABA is produced by epithelial, endocrine, immune, and hepatic cells, where it functions through both receptor-dependent and metabolic pathways [2,3].

In addition to signaling through GABA_A_ and GABA_B_ receptors, GABA participates in the GABA shunt, which links amino acid metabolism to mitochondrial function, intermediary carbon flux, and redox balance [3,4]. In the pulmonary and gastrointestinal tracts, local GABA signaling influences barrier integrity, immune tone, enteroendocrine function, and gut–brain communication [5,6,7]. GABA is also present in natural and fermented foods and can be produced in situ by members of the intestinal microbiota [8,9,10].

These characteristics distinguish GABA from classical essential nutrients defined by deficiency syndromes. In this review, GABA is therefore framed as a nonessential functional nutrient: an endogenously synthesized metabolic-signaling molecule whose dietary or microbial availability may modulate physiological regulation under specific conditions. This framing is relevant to nutrition science because it places GABA at the intersection of diet, microbiota, metabolism, and host signaling.

This narrative review is tailored to the scope of nutrients and focuses on five major themes: (i) exogenous sources of GABA, including dietary intake and intestinal microbial production; (ii) intestinal absorption, hepatic uptake, and metabolic fate; (iii) peripheral GABA signaling and the biological activities of GABA-derived metabolites; (iv) experimental, preclinical, and clinical evidence for GABA supplementation in immune, endocrine, and metabolic regulation; and (v) key knowledge gaps and future directions relevant to GABA-based nutritional strategies.

## 2. Literature Search Strategy

The literature included in this narrative review was identified primarily through structured searches of the PubMed database, complemented by manual screening of reference lists from relevant reviews and primary research articles. The search strategy was designed to capture both foundational and contemporary literature addressing the biological roles and supplementation potential of GABA as a functional nutrient. Search terms included “γ-aminobutyric acid,” “GABA,” “functional nutrient,” “gut microbiota,” “hepatic metabolism,” “immune regulation,” “endocrine signaling,” and “metabolic regulation,” along with relevant combinations of these terms. Where appropriate, additional topic-specific terms were used to refine retrieval within specific domains, including intestinal absorption, GABA shunt metabolism, receptor signaling, and microbial or probiotic GABA production.

The search encompassed mechanistic studies and available preclinical and clinical investigations published from 2000 to 2025. Seminal studies published prior to 2000 were also included when necessary to provide foundational insights into GABA metabolism, receptor biology, and peripheral signaling mechanisms. The more recent literature was emphasized when it provided advances in microbiota-related mechanisms, systemic metabolic integration, and translational outcomes. Although structured search terms and eligibility considerations were used to guide literature identification, this review was not conducted according to PRISMA guidelines and should not be interpreted as a systematic review.

Given the broad and integrative scope of this review, study selection was guided by thematic and scientific relevance rather than predefined inclusion and exclusion criteria. Articles were prioritized based on their ability to inform key thematic areas, including exogenous sources, absorption and hepatic handling, signaling mechanisms, and functional effects in immune, endocrine, and metabolic contexts. Emphasis was placed on mechanistic and influential studies, with human investigations included where available to provide translational contexts. Emphasis was placed on mechanistic studies, influential preclinical investigations, and available human studies that provided translational insight into the physiological effects of GABA supplementation.

Additional relevant articles were identified through reference-list screening. In general, this narrative review reflects an interpretive and integrative synthesis intended to provide a coherent overview of the field rather than an exhaustive systematic assessment of all published studies. Table 1 summarizes literature search and study selection. Evidence was organized according to biological domain (immune, endocrine, epithelial, hepatic, metabolic, and microbiota-related) and level of evidence (cellular, animal, and human studies) to facilitate comparison of mechanistic, preclinical, and clinical findings.

## 3. Exogenous Sources, Bioavailability, and Metabolic Fate of GABA

### 3.1. Dietary Sources of GABA

GABA is naturally present in a range of plant foods, although its concentration varies according to species, cultivar, environmental conditions, and postharvest handling. Vegetables such as tomatoes, spinach, potatoes, sweet potatoes, broccoli, and soybeans contain measurable amounts of GABA, but the concentrations observed in unprocessed foods are generally modest [11]. In plants, GABA is synthesized primarily through decarboxylation of glutamate through the enzymatic activity of glutamate decarboxylase (GAD), and its production is rapidly upregulated by environmental stressors such as hypoxia, mechanical damage, salinity, and temperature change [12,13].

Cereal grains also contain GABA, with whole grains typically containing more GABA than refined products because of localization within bran and germ fractions [11]. Brown rice contains more GABA than polished white rice, but the absolute amount remains limited unless additional processing is used [11]. As a result, interest in increasing dietary GABA has shifted toward food-processing approaches that substantially enrich GABA content.

Germination and sprouting are effective strategies for increasing GABA concentrations in plant foods. During germination, endogenous GAD activity is activated, thereby accelerating glutamate-to-GABA conversion [11]. Germinated brown rice (“GABA rice”) is the best-studied example, although the extent of enrichment depends on soaking time, pH, temperature, and cultivar [11].

Fermented foods constitute some of the richest dietary sources of GABA. During fermentation, microorganisms, particularly lactic acid bacteria, decarboxylate free glutamate within the food matrix and can substantially increase GABA content [14,15]. GABA-enriched fermented foods include vegetables, dairy products, soy-based foods, cereals, and beverages [14,15]. However, variability remains high and depends on microbial strain, substrate availability, fermentation duration, and pH.

### 3.2. Intestinal Microbial Production of GABA

In addition to host synthesis, the intestinal microbiota is a major source of luminal GABA and may influence host physiology. Multiple commensal taxa, including species within *Bacteroides*, *Lactobacillus*, and *Bifidobacterium*, possess glutamate decarboxylase systems capable of converting glutamate to GABA [8,9]. Metagenomic and metabolomic studies have also identified GABA-producing species in *Parabacteroides*, *Enterococcus*, *Clostridium*, and *Escherichia* [10].

Among these taxa, *Bacteroides* species appear to be especially important contributors. Genomic analyses indicate that many human-derived *Bacteroides* strains harbor complete GAD systems, and several produce substantial amounts of GABA in vitro, particularly under acidic conditions [16]. Lactic acid bacteria and *Bifidobacteria* are also of interest because of their relevance to fermented foods and probiotic development [9,10].

Microbial GABA production is strongly shaped by the local luminal environment. Low pH, osmotic stress, and high glutamate availability can all increase microbial GAD activity because decarboxylation consumes intracellular protons [8,16]. Luminal GABA concentrations therefore reflect a balance between production and consumption, as some microbial taxa can further metabolize GABA as a carbon or nitrogen source. Consequently, the presence of GABA-producing taxa alone is not sufficient to predict intestinal GABA availability.

Microbiota-derived GABA may influence epithelial cells, immune cells, enteric neurons, and vagal afferents, thereby modulating visceral sensitivity, barrier integrity, endocrine signaling, and neuroimmune communication [7,10]. In animal models, administration of GABA-producing *Bifidobacterium* or *Lactobacillus* strains can alter visceral pain-related phenotypes, stress-associated behaviors, and selected metabolic readouts [17]. Although direct causal evidence in humans remains limited, these data support the concept that microbial GABA may function as a postbiotic mediator at the intestinal interface [10].

### 3.3. Intestinal Absorption of GABA

GABA is a small, highly polar, zwitterionic amino acid with low lipophilicity. These properties make substantial passive transcellular diffusion across the intestinal epithelium unlikely, implying that carrier-mediated transport is the predominant route of absorption [18]. The clearest mechanistic evidence derives from human intestinal epithelial models, in which GABA is transported across Caco-2 monolayers through a proton-coupled, sodium-independent mechanism consistent with proton-coupled amino acid transporter 1 (PAT1/SLC36A1) [18]. This transport is pH dependent, electrogenic, and competitively inhibited by certain small amino acid analogues [18].

Studies indicate that GABA is absorbed mainly in the small intestine, particularly in jejunal and ileal regions where PAT1 expression is highest [19]. Overall absorption may also be influenced by meal composition and luminal pH [18]. Once absorbed, GABA enters the portal circulation and is delivered directly to the liver.

### 3.4. Hepatic Uptake, Metabolism, and First-Pass Handling

After entering the portal circulation, GABA is efficiently taken up by hepatocytes. GABA transporter 2 (GAT2/SLC6A13) is highly expressed on the sinusoidal membrane of periportal hepatocytes and appears to be functionally important for hepatic GABA handling [3,20]. Despite substantial first-pass hepatic extraction, orally administered GABA can still be detected in systemic circulation. High-performance liquid chromatographic analyses report basal plasma GABA concentrations of approximately 50–100 ng/mL in healthy volunteers [21,22], indicating the presence of a relatively low but stable circulating GABA pool under physiological conditions. Notably, oral administration of a single 2.0 g dose of GABA increased plasma concentrations approximately 10–20-fold, reaching 1000–1200 ng/mL within 0.5–1 h (*T_max_*), with an elimination half-life of approximately 5 h and no apparent accumulation following repeated dosing [22]. These findings demonstrate that orally administered GABA can substantially increase systemic GABA levels despite efficient hepatic first-pass uptake.

The liver is not only a sink for circulating GABA, but also a site of endogenous GABA signaling and production. Hepatocytes express GABA receptor sites and both hepatocytes and cholangiocytes express GAD isoforms [23]. Under some metabolic conditions, especially obesity and hepatic lipid accumulation, endogenous hepatic GABA production may exceed the contribution of dietary GABA [3,24]. This distinction is biologically important because local hepatic GABA may influence vagal signaling, insulin sensitivity, and energy balance independently of oral supplementation [24,25].

Within hepatocytes, GABA is metabolized through the GABA shunt. GABA transaminase (GABA-T) converts GABA to succinic semialdehyde, which is subsequently converted to succinate by succinic semialdehyde dehydrogenase [3,4]. In this way, GABA catabolism is linked directly to the tricarboxylic acid (TCA) cycle and broader intermediary metabolism [3,4]. Because hepatic uptake and metabolism are efficient, a considerable fraction of orally absorbed GABA likely undergoes first-pass extraction, which limits systemic exposure relative to the absorbed dose [3,22].

## 4. Mechanisms of Peripheral GABA Signaling

### 4.1. GABA_A_ and GABA_B_ Receptors in Peripheral Tissues

GABA_A_ receptors are pentameric ligand-gated chloride channels assembled from multiple subunits, including α1-6, β1-3, γ1-3, δ, ε, θ, π, and ρ1-3 families [26,27,28,29]. GABA_B_ receptors are obligate heterodimers composed of GABBR1 and GABBR2 and signal through Gi/o proteins to inhibit adenylyl cyclase, reduce cAMP, activate potassium channels, and inhibit voltage-gated calcium channels [30]. In contrast to mature CNS neurons, where GABA_A_ receptor activation typically causes membrane hyperpolarization through Cl^−^ influx, the effects of GABA_A_ receptor activation in peripheral cells depend on cell-specific chloride gradients and electrophysiological properties.

**Figure 1:**
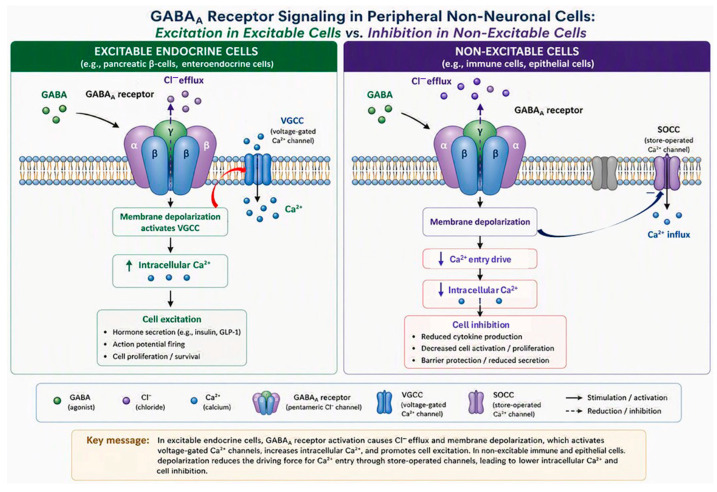
Cell-type dependent effects of GABA_A_ receptor activation in peripheral non-neuronal cells. In certain excitable endocrine cells, such as pancreatic β-cells, GABA_A_ receptor stimulation by GABA causes Cl^−^ efflux and membrane depolarization, leading to activation of voltage-gated Ca^2+^ channels and increased intracellular Ca^2+^ signaling. In non-excitable immune and epithelial cells, however, membrane depolarization reduces the electrochemical driving force for Ca^2+^ entry through store-operated Ca^2+^ channels, thereby attenuating Ca^2+^-dependent responses. This model illustrates how GABA_A_ receptor activation induces cell excitation or inhibition depending on cellular electrophysiological context.

The biological consequences of GABA receptor activation outside the CNS are highly cell type dependent. As illustrated in Figure 1, in most excitable endocrine cells such as pancreatic β-cells but not α-cells, GABA_A_ receptor activation causes membrane depolarization and voltage-gated calcium channel activation thus increasing calcium-dependent hormone secretion. In many immune or epithelial contexts, however, GABA-induced depolarization results in reduction of calcium entry and cytokine secretion hence anti-inflammatory effects [31,32]. Therefore, peripheral GABA signaling should not be interpreted as uniformly inhibitory or excitatory across tissues.

### 4.2. Endocrine, Immune, Epithelial, and Hepatic GABA Signaling

Endocrine tissues provide some of the clearest examples of peripheral GABA signaling. In pancreatic islets, β-cells synthesize and release GABA, which then acts through autocrine and paracrine mechanisms to promote β-cell survival and insulin secretion, but to inhibit α-cell proliferation and glucagon secretion [33,34,35]. Reduced intra-islet GABA signaling contributes to β-cell apoptosis and α-cell expansion under diabetic conditions [34].

Immune cells also possess GABAergic machinery. T cells, B cells, macrophages, dendritic cells, and antigen-presenting cells express functional GABA receptors as well as enzymes and transporters involved in GABA synthesis and handling [2,36,37]. GABA signaling in these cells is typically associated with suppression of inflammatory cytokine production, reduced effector activation, and shifts toward regulatory phenotypes [36,38,39,40].

Epithelial cells in the lung and intestine also express GABA receptors and GABA-synthesizing enzymes [5,6,41]. In these tissues, GABA signaling influences transepithelial ion transportation, water movement, cytokine secretion, and tight-junction regulation, thereby contributing to barrier integrity and mucosal homeostasis [7,42,43]. Hepatocytes and cholangiocytes similarly engage in GABAergic signaling relevant to tissue injury, regeneration, and metabolic control [3,23].

### 4.3. GABA-Derived Metabolites and Mitochondrial Integration

Because GABA is metabolized through the GABA shunt, receptor-mediated signaling is only part of its physiological relevance. GABA turnover contributes to mitochondrial carbon flux, ATP generation, redox state, and metabolite signaling [3,4]. One important product of GABA metabolism is succinate, which functions as both a TCA-cycle intermediate and a signaling metabolite [43,44,45,46]. Through succinate receptor 1 (SUCNR1)-dependent and independent pathways, succinate can influence inflammatory responses, renin signaling, and hepatic steatosis [43,44,45]. Thus, the biological consequences of succinate signaling may be either adaptive or pathogenic depending on context. This metabolic dimension is especially relevant in liver, pancreas, and immune cells, where GABA turnover may influence not only signaling but also cellular bioenergetics. Accordingly, GABA should be considered both a signaling molecule and a metabolically active intermediary.

The mechanistic studies discussed above demonstrate that peripheral GABA signaling extends well beyond the nervous system. Table 2 summarizes representative studies illustrating the diversity of GABA actions across intestinal, immune, endocrine, epithelial, hepatic, and microbiota-related pathways.

## 5. Evidence for Supplemental GABA in Immune, Endocrine, and Metabolic Regulation

### 5.1. Immune Modulation and Barrier Regulation

Experimental studies consistently indicate that activating GABA signaling dampens proinflammatory responses. GABA_A_ receptors are expressed on epithelial barriers and on major innate and adaptive immune cell populations, including T cells, B cells, macrophages, dendritic cells, and antigen-presenting cells [2,5,36,41]. In multiple preclinical systems, increasing GABA reduces intracellular calcium signaling, suppresses secretion of pro-inflammatory cytokines, and limits pathogenic immune activation [2,36].

Within innate immunity, monocytes, macrophages, and dendritic cells possess a conserved GABAergic machinery integrating synthesis, secretion, receptor activation, and calcium signaling [2,37]. Endogenous GABA released from B cells has also been reported to redirect macrophages toward more regulatory phenotypes [38]. In adaptive immunity, GABA signaling suppresses T-cell proliferation and can shift helper-cell differentiation away from pro-inflammatory Th17-like states [39,47,48].

These immunological effects extend to epithelial barriers. In pulmonary and intestinal epithelial cells, local GABA signaling influences ion and water transport, cytokine secretion, and tight-junction biology [5,6,41,42]. In this way, GABA contributes to barrier homeostasis in addition to immune regulation. However, intestinal biology is complex. Whereas some studies support barrier protection, others report that GABA_A_ receptor activation in the colon can worsen epithelial dysfunction in certain inflammatory contexts [51,52]. Therefore, the tissue context, receptor localization, and disease state all require careful consideration.

Preclinical studies also suggest that GABA administration can attenuate disease severity in several autoimmune or inflammatory disorders, including experimental autoimmune encephalomyelitis, rheumatoid arthritis, and some forms of colitis [36,51,62]. Nevertheless, human translational evidence remains limited and does not yet establish durable therapeutic benefit in defined immune-mediated diseases.

### 5.2. Endocrine Regulation and Integration

Pancreatic islets represent one of the best-characterized peripheral GABA systems. β-cells synthesize and release GABA, and both β-cells and α-cells express functional GABA receptors. GABA fosters β-cell function and survival and inhibits glucagon secretion and restrains α-cell proliferation through autocrine and paracrine mechanisms [32,33,34,52]. In preclinical diabetes models, supplemental GABA can preserve β-cell mass, reduce inflammatory injury in islets, and improve glycemic control [31,34,63]. Some studies have reported that GABA administration increases β-cell mass by promoting α-to-β cell conversion [64]. However, evidence supporting α-to-β transdifferentiation remains controversial and has not been consistently reproduced across experimental systems. Whether these effects result from direct cellular reprogramming or indirect mechanisms involving β-cell proliferation and survival remains controversial [65] and continues to be actively investigated.

Human studies are more limited, but are not absent. In healthy individuals, oral GABA increases circulating insulin and glucagon under fasting and fed conditions without acutely lowering blood glucose [22]. Thus, oral GABA is biologically active in humans, although its clinical efficacy remains uncertain.

At the gut level, GABA may interact with enteroendocrine and vagal pathways. Experimental studies indicate that GABA can stimulate GLP-1 release in intestinal L-cell models [53]. GLP-1 is a known activator of vagal afferent pathways involved in satiation and postprandial metabolic signaling [66,67]. Consistent with this framework, oral, but not intraperitoneal, GABA has been reported to potentiate postprandial vagal afferent activation in experimental settings [54]. These findings support the view that the intestinal interface is a key site of action for oral GABA.

Hepatic GABA signaling is more context dependent. Endogenous hepatic GABA production has been linked to insulin resistance and altered vagal signaling under metabolic stress [24,25], whereas systemic GABA administration or preservation of endogenous GABA signaling has been associated with improved outcomes in other experimental contexts [23,68,69]. These apparently divergent observations likely reflect differences between localized hepatic signaling and whole-body GABA exposure.

### 5.3. Glucose and Lipid Metabolism

A substantial portion of the preclinical literature suggests that GABA supplementation can influence systemic metabolism. In experimental models, GABA has been reported to improve glucose tolerance, enhance insulin sensitivity, increase hepatic glycogen storage, promote peripheral glucose uptake, and reduce hepatic gluconeogenesis [55,56,57]. These effects probably reflect the combined influence of reduced inflammation, altered hepatic metabolism, improved islet function, and modified neural signaling.

GABA has also been reported to influence lipid metabolism and body composition. Animal studies describe reductions in hepatic lipid accumulation, improvements in circulating lipid profiles, and attenuation of diet-induced weight gain [57,58,59,60]. Recent experimental work also suggests that GABA may alter energy partitioning toward growth-related pathways while reducing adipose storage [61].

However, these metabolic findings should be interpreted cautiously in relation to humans. Human intervention studies have shown endocrine effects and favorable short-term tolerability, but they have not consistently demonstrated robust improvements in glucose homeostasis or body composition across populations [22,70,71]. Therefore, the metabolic potential of GABA in humans remains plausible but incompletely established.

### 5.4. Microbiota-Related and Probiotic Contexts

GABA-producing probiotics provide an additional line of preclinical evidence linking GABA biology to host physiology. In mouse models, GABA-producing *Lactobacillus* or *Bifidobacterium* strains have attenuated inflammatory responses, altered stress-related behavior, and improved selected metabolic readouts [17]. These findings support the concept that microbiota-derived GABA may complement host or supplemental GABA at the intestinal interface. At present, however, the causal and quantitative contributions of microbial GABA to human physiology remain poorly resolved.

Animal studies have examined the effects of GABA supplementation across a broad range of immune, endocrine, metabolic, and hepatic conditions. Table 3 summarizes representative in vivo studies and their principal findings.

Compared with the extensive mechanistic and animal literature, human studies remain relatively limited in number and scope. Table 4 summarizes representative clinical and translational studies evaluating oral GABA supplementation and highlights the current gap between biological activity and demonstrated clinical efficacy.

## 6. Discussion

### 6.1. Mechanistic Studies Supporting Peripheral GABA Biology

GABA functions beyond its established role as a neurotransmitter and participates in a wide range of physiological processes in peripheral tissues. Studies have identified GABA transport systems in the intestine and liver, receptor-mediated signaling pathways in epithelial, endocrine, and immune cells, and metabolic functions linked to the GABA shunt [2,3,4,18,19,20,33,34,35,36,37,38,39].

Peripheral GABA signaling has been implicated in immune regulation, pancreatic islet homeostasis, epithelial barrier function, hepatic metabolism, and host–microbiota interactions [2,3,7,8,9,10,23,33,34,35,36,37,38,39]. Endocrine and immune mechanisms have been studied most extensively [31,32,33,34,35,36,37,38,39,47,48], whereas the contribution of microbiota-derived GABA and GABA-derived metabolites to human physiology remains less clear [8,9,10,16,43,44,45,46].

GABA also functions as a metabolic intermediate through the GABA shunt. Its metabolism generates downstream products that influence mitochondrial function, energy metabolism, and inflammatory signaling pathways [3,4,43,44,45,46].

### 6.2. Translational Gap Between Preclinical and Clinical Data

Experimental evidence supporting GABA supplementation is extensive. Animal studies have reported beneficial effects in models of diabetes, obesity, inflammation, autoimmune disease, and liver injury [23,31,36,63,68,72]. Improvements in glucose tolerance, insulin sensitivity, β-cell preservation, inflammatory regulation, and lipid metabolism have been observed across multiple experimental systems [31,36,63,72].

Human studies show that oral GABA is absorbed, increases circulating GABA concentrations, and affects endocrine responses, including insulin and glucagon secretion [22]. However, evidence for clinically meaningful benefits in metabolic disease, obesity, or immune-mediated disorders remains sparse [70,71]. Most studies have been small and have focused on short-term physiological outcomes rather than clinical endpoints [22,70,71]. Larger randomized controlled trials are needed before conclusions can be drawn regarding the therapeutic or preventive value of GABA supplementation.

### 6.3. Context-Dependent Nature of GABA Signaling

The effects of GABA signaling vary across tissues and physiological settings. Responses depend on cell type, receptor expression, chloride gradients, metabolic state, and disease context [26,27,28,29,30]. This variability is evident in epithelial biology. Several studies indicate that GABA contributes to epithelial homeostasis and barrier function [6,41,42]. In experimental colitis models, GABA treatment has reduced visceral hypersensitivity and inflammation [52], whereas activation of specific receptor pathways can worsen epithelial dysfunction under certain inflammatory conditions [51].

A similar pattern is observed in the liver. Increased endogenous hepatic GABA production has been linked to insulin resistance and altered metabolic regulation in obesity [24,25], whereas exogenous GABA administration has shown protective effects in models of acute and ethanol-induced liver injury [23,68]. These observations indicate that the physiological effects of GABA depend on the biological context in which signaling occurs. Such variability is consistent with the concept of GABA as a functional nutrient rather than a uniformly beneficial supplement.

### 6.4. Current Knowledge Gaps and Methodological Challenges

Several uncertainties limit interpretation of the current literature. First, dose–response relationships remain poorly defined. Animal studies have employed a wide range of supplementation strategies and experimental designs [31,55,56,57,58,59,60,61,63,72], making comparisons difficult and limiting extrapolation to human dietary exposure.

Second, important pharmacokinetic questions remain unresolved. Although intestinal absorption and hepatic uptake pathways have been identified [18,19,20,22], little is known regarding tissue-specific exposure following oral supplementation. Because substantial first-pass hepatic extraction occurs [3,20,22], circulating concentrations may not reflect local tissue exposure or target engagement.

Third, the physiological importance of microbiota-derived GABA remains uncertain. Experimental evidence suggests roles in epithelial, immune, and neuroendocrine regulation [7,8,9,10,16], but its quantitative contribution to human physiology remains unknown.

Fourth, sex-specific responses have received limited attention. Given established sex-related differences in immune regulation, metabolism, and microbiota composition [61,74], future studies should evaluate biological responses in males and females separately.

**Figure 2:**
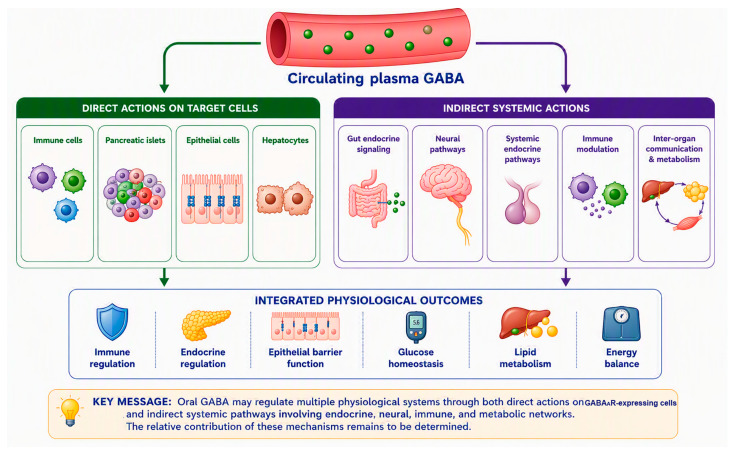
Proposed conceptual model of the direct and indirect mechanisms underlying the physiological effects of oral GABA supplementation. Following intestinal absorption and partial hepatic first-pass metabolism, GABA may influence physiological function through both direct actions on immune, endocrine, epithelial, and hepatic cells and indirect systemic mechanisms involving endocrine responses, incretin secretion, gut–brain communication, vagal pathways, and inter-organ signaling networks. These pathways may converge to regulate immune function, endocrine homeostasis, epithelial barrier integrity, glucose metabolism, lipid metabolism, and whole-body energy balance. The relative contributions of these mechanisms remain incompletely understood.

Finally, long-term human studies are lacking. Most investigations have examined acute administration or short intervention periods [22,70]. Consequently, long-term efficacy, safety, and sustainability remain insufficiently characterized. The physiological effects of oral GABA likely involve interactions among receptor-mediated, endocrine, neural, and metabolic pathways, but the relative contribution of these mechanisms remains uncertain. Figure 2 summarizes a conceptual framework for these interactions.

### 6.5. Strengths and Limitations of the Present Review

This review integrates evidence from nutrition, microbiology, metabolism, immunology, endocrinology, and physiology to examine GABA as a functional nutrient. By considering mechanistic, preclinical, and clinical studies together [2,3,4,5,6,7,8,9,10,22,23,24,25,31,32,33,34,35,36,37,38,39,40,41,42,43,44,45,46,47,48,49,50,51,52,53,54,55,56,57,58,59,60,61,62,63,70,71,72,73], it highlights both the breadth of peripheral GABA biology and the challenges involved in translating experimental findings into clinical applications.

Several limitations should be acknowledged. This was a narrative review and did not follow a predefined systematic-review protocol or PRISMA methodology. Study selection was guided by thematic relevance rather than exhaustive literature capture. In addition, heterogeneity among experimental models, supplementation protocols, outcome measures, and study populations limits direct comparison between studies [22,70,71,73].

## 7. Conclusions and Future Directions

The literature supports GABA as a biologically active functional nutrient involved in immune, endocrine, epithelial, hepatic, and metabolic regulation. Experimental studies consistently demonstrate beneficial effects across multiple disease models, whereas human studies remain limited and have not established clear clinical efficacy [3,4,23,31,36,43,44,45,46,63,68,72]. The physiological effects of GABA appear to depend on tissue, metabolic status, and disease context [24,25,51,52]. Future studies should prioritize well-powered clinical trials, dose–response analyses, long-term safety assessments, and biomarkers of tissue-specific GABA exposure and target engagement [8,9,10,22,70,71,74]. 

Future research should address four areas. First, adequately powered randomized controlled trials are needed to determine whether the biological effects of GABA translate into clinical benefits in metabolic, inflammatory, and immune-mediated conditions. Second, biomarkers of tissue-specific GABA exposure and target engagement are needed to identify responders and distinguish hepatic, intestinal, endocrine, and immune mechanisms of action. Third, sex-specific responses should be evaluated prospectively, given emerging evidence for differences in endocrine and immune regulation [61,74]. Finally, improved approaches are needed to quantify the relative contributions of dietary, host-derived, and microbiota-derived GABA pools in humans. Addressing these questions will help clarify the physiological and clinical relevance of GABA supplementation.

## Figures and Tables

**Table 1:** Literature search and study selection framework.

Category	Description
Database	PubMed.
Search period	January 2000 to March 2025, supplemented by selected landmark studies published before 2000 where relevant.
Study types	In vitro, animal, observational human, and clinical intervention studies.
Topics	Dietary and microbial sources of GABA, absorption, hepatic metabolism, peripheral signaling, immune regulation, endocrine regulation, barrier function, and metabolic effects.
Literature selection considerations	Studies investigating GABA biology, metabolism, microbial production, signaling mechanisms, or supplementation-related physiological outcomes.
Literature exclusion considerations	Conference abstracts, duplicate reports, studies lacking sufficient methodological detail, and articles focused exclusively on CNS neurotransmission without nutritional or peripheral physiological relevance.
Evidence synthesis	Evidence was organized according to mechanistic domain and level of studies (in vitro, animal, or human).

**Table 2:** Representative mechanistic evidence supporting peripheral GABA biology.

Mechanistic Domain	Experimental Model	Mechanism	Principal Finding	Ref.
Intestinal absorption	Caco-2 epithelial cells	PAT1-mediated transport	GABA absorption occurs primarily through carrier-mediated transport	[18,19]
Hepatic metabolism	Hepatocytes	GAT-mediated uptake and GABA shunt metabolism	Hepatic uptake contributes to first-pass extraction and integration into intermediary metabolism	[3,4,20]
Immune regulation	T lymphocytes	GABA_A_ receptor activation	Suppresses T-cell proliferation and inflammatory responses	[31,47,48]
Immune regulation	Experimental autoimmune encephalomyelitis model	GABAergic signaling	Reduces autoimmune inflammation and disease severity	[36,49]
Immune regulation	Pulmonary macrophages	Autocrine GABA signaling	Regulates macrophage activation phenotype and inflammatory responses	[37]
Immune regulation	B cell–macrophage interactions	Endogenous GABA production	Promotes regulatory IL-10+ macrophage phenotypes	[38]
Endocrine regulation	Pancreatic β-cells	Autocrine GABA signaling	Modulates insulin secretion and β-cell function	[33,34,35]
Endocrine regulation	Pancreatic α-cells	Paracrine GABA signaling	Suppresses glucagon secretion and limits α-cell proliferation	[34,50]
Epithelial barrier regulation	Intestinal epithelial cells	GABAA receptor signaling	Regulates transport and fluid secretion	[41]
Epithelial barrier regulation	Intestinal injury models	GABA_A_ receptor/AMPK-autophagy pathway	Reduces epithelial apoptosis and supports barrier integrity	[42]
Epithelial barrier regulation	Colitis models	GABA receptor signaling	Demonstrates context-dependent barrier-protective and barrier-disruptive effects	[51,52]
Gut–endocrine signaling	GLP-1 secreting intestinal L-cells	GABA-stimulated hormone release	Enhances GLP-1 secretion and gut signaling pathways	[53]
Gut–brain communication	Animal feeding studies	Vagal afferent activation	Oral GABA enhances postprandial vagal afferent activation and increases satiation	[54]
Metabolic regulation	High-fat diet mouse models	Immune and endocrine modulation	Improves glucose tolerance and insulin sensitivity	[55,56,57]
Lipid metabolism	Obese mouse models	Modulation of adipose and hepatic metabolism	Reduces adiposity and hepatic lipid accumulation	[58,59,60,61]
Microbiota-host interactions	Bacteroides, *Lactobacillus*, *Bifidobacterium* spp.	Microbial GABA production	Establishes microbiota-derived GABA as a potential postbiotic mediator	[8,9,10,16]
Metabolite-mediated signaling	GABA shunt metabolism	Succinate generation	Links GABA metabolism with mitochondrial bioenergetics and inflammatory signaling pathways	[4,43,44,45,46]

**Table 3:** Representative animal studies evaluating GABA supplementation.

Evidence Domain	Study Model	Intervention	Principal Findings	Ref.
Immune	Type 1 diabetes mouse model	Oral GABA	Reduced T-cell-mediated inflammation and delayed disease progression	[31]
Immune	Rheumatoid arthritis mouse model	Oral GABA	Reduced inflammatory responses and disease severity	[72]
Immune	Experimental autoimmune encephalomyelitis	GABA treatment	Attenuated disease severity and inflammatory responses	[36,49]
Immune	Pulmonary macrophage model	GABAergic signaling	Modulated macrophage phenotype and inflammatory responses	[37]
Endocrine	Type 1 diabetes mouse model	GABA supplementation	Promoted β-cell survival and regeneration; improved diabetes outcomes	[63]
Endocrine	Mouse model of type 1 diabetes	Endogenous/paracrine GABA signaling	Suppressed α-cell proliferation and preserved islet homeostasis	[34]
Endocrine	Pancreatic islet models	GABA signaling	Modulated β-cell function and glucose-dependent insulin secretion	[33]
Metabolic	High-fat diet mouse model	Oral GABA	Improved glucose tolerance and insulin sensitivity; reduced inflammation	[55]
Metabolic	Type 2 diabetic rat offspring	GABA administration	Improved insulin sensitivity and hepatic metabolic parameters	[57]
Metabolic	Type 2 diabetic mouse model fed GABA-rich yogurt	Dietary GABA enrichment	Improved insulin sensitivity and glucose control	[56]
Metabolic	High-fat diet-induced obesity model	GABA supplementation	Reduced body weight gain and adipose accumulation	[58]
Metabolic	Obese mouse model	GABA-mediated microbiota modulation	Altered gut microbiota and promoted beige adipocyte formation	[59]
Metabolic	High-fat diet-induced obesity model	GABA-enriched fermented extract	Reduced obesity-associated metabolic dysfunction	[60]
Metabolic	Adolescent mouse model	Long-term GABA supplementation	Altered food intake, growth, ghrelin signaling, and lipid metabolism	[61]
Hepatic	Acute liver injury rat model	GABA administration	Reduced hepatic injury and enhanced hepatoprotection	[23]
Hepatic	Ethanol-induced liver injury model	GABA administration	Protected against ethanol-induced hepatic injury	[68]
Metabolic	High-fat diet obese mice	Peripheral GABA plus GABA-T inhibition	Enhanced weight-loss and appetite-regulatory effects	[69]

**Table 4:** Representative human and translational studies relevant to oral GABA supplementation.

Study Domain	Study Population	Study Design	Intervention	Principal Findings	Ref.
Endocrine	Healthy adults	Clinical pharmacokinetic/pharmacodynamic study	Oral GABA	Oral GABA increased circulating GABA, insulin, and glucagon levels without substantially altering blood glucose	[22]
Endocrine/Metabolic	Adults with prediabetes	Randomized, placebo-controlled trial	Oral GABA supplementation	Demonstrated modest effects on glucose regulation, with limited evidence for clinically meaningful benefit	[70]
Endocrine	Children with newly diagnosed type 1 diabetes	Randomized clinical trial	Oral GABA alone or in combination with GAD therapy	Provided evidence of biological activity, although clinical efficacy remained inconclusive.	[71]
Endocrine	Human pancreatic islets (ex vivo translational)	Human mechanistic study	Endogenous GABA signaling	Confirmed physiological importance of GABA signaling in human islets	[35,73]

## Data Availability

No new data were created or analyzed in this study. Data Sharing is not applicable to this article.

## References

[1] Huang D., Alexander P.B., Li Q.-J., Wang X.-F. (2023). GABAergic signaling beyond synapses: An emerging target for cancer therapy. Trends Cell Biol..

[2] Bhandage A.K., Barragan A. (2021). GABAergic signaling by cells of the immune system: More the rule than the exception. Cell. Mol. Life Sci..

[3] Kim K., Yoon H. (2023). Gamma-aminobutyric acid signaling in damage response, metabolism, and disease. Int. J. Mol. Sci..

[4] Kumrungsee T. (2024). Is hepatic GABA transaminase a promising target for obesity and epilepsy treatments?. Biosci. Biotechnol. Biochem..

[5] Xiang Y.-Y., Wang S., Liu M., Hirota J.A., Li J., Ju W., Fan Y., Kelly M.M., Ye B., Orser B. (2007). A GABAergic system in airway epithelium is essential for mucus overproduction in asthma. Nat. Med..

[6] Deng Z., Li D., Yan X., Lan J., Han D., Fan K., Chang J., Ma Y. (2023). Activation of GABA receptor attenuates intestinal inflammation by modulating enteric glial cells function through inhibiting NF-κB pathway. Life Sci..

[7] Conn K.A., Borsom E.M., Cope E.K. (2024). Implications of microbe-derived γ-aminobutyric acid (GABA) in gut and brain barrier integrity and GABAergic signaling in Alzheimer’s disease. Gut Microbes.

[8] Strandwitz P., Kim K.H., Terekhova D., Liu J.K., Sharma A., Levering J., McDonald D., Dietrich D., Ramadhar T.R., Lekbua A. (2019). GABA-modulating bacteria of the human gut microbiota. Nat. Microbiol..

[9] Duranti S., Ruiz L., Lugli G.A., Tamés H., Milani C., Mancabelli L., Mancino W., Longhi G., Carnevali L., Sgoifo A. (2020). *Bifidobacterium adolescentis* as a key member of the human gut microbiota in the production of GABA. Sci. Rep..

[10] Braga J.D., Thongngam M., Kumrungsee T. (2024). Gamma-aminobutyric acid as a potential postbiotic mediator in the gut–brain axis. npj Sci. Food.

[11] Pencheva D., Teneva D., Denev P. (2023). Validation of HPLC method for analysis of gamma-aminobutyric and glutamic acids in plant foods and medicinal plants. Molecules.

[12] Hu Y., Huang X., Xiao Q., Wu X., Tian Q., Ma W., Shoaib N., Liu Y., Zhao H., Feng Z. (2024). Advances in plant GABA research: Biological functions, synthesis mechanisms and regulatory pathways. Plants.

[13] Yuan D., Wu X., Gong B., Huo R., Zhao L., Li J., Lü G., Gao H. (2023). GABA metabolism, transport and their roles and mechanisms in the regulation of abiotic stress (hypoxia, salt, drought) resistance in plants. Metabolites.

[14] Sahab N.R.M., Subroto E., Balia R.L., Utama G.L. (2020). γ-Aminobutyric acid found in fermented foods and beverages: Current trends. Heliyon.

[15] Cataldo P.G., Villena J., Elean M., Savoy de Giori G., Saavedra L., Hebert E.M. (2020). Immunomodulatory properties of a γ-aminobutyric acid-enriched strawberry juice produced by *Levilactobacillus brevis* CRL 2013. Front. Microbiol..

[16] Otaru N., Ye K., Mujezinovic D., Berchtold L., Constancias F., Cornejo F.A., Krzystek A., de Wouters T., Braegger C., Lacroix C. (2021). GABA production by human intestinal *Bacteroides* spp.: Prevalence, regulation, and role in acid stress tolerance. Front. Microbiol..

[17] Patterson E., Ryan P.M., Cryan J.F., Dinan T.G., Ross R.P., Fitzgerald G.F., Stanton C. (2016). Gut microbiota, obesity and diabetes. Postgrad. Med. J..

[18] Thwaites D.T., Basterfield L., McCleave P.M., Carter S.M., Simmons N.L. (2000). Gamma-aminobutyric acid (GABA) transport across human intestinal epithelial (Caco-2) cell monolayers. Br. J. Pharmacol..

[19] Chen Z., Fei Y.-J., Anderson C.M.H., Wake K.A., Miyauchi S., Huang W., Thwaites D.T., Ganapathy V. (2003). Structure, function and immunolocalization of a proton-coupled amino acid transporter (hPAT1) in the human intestinal cell line Caco-2. J. Physiol..

[20] Tachikawa M., Ikeda S., Fujinawa J., Hirose S., Akanuma S., Hosoya K.-I. (2012). γ-Aminobutyric acid transporter 2 mediates the hepatic uptake of guanidinoacetate, the creatine biosynthetic precursor, in rats. PLoS ONE.

[21] Petty F., Kramer G.L., Fulton M., Moeller F.G., Rush A.J. (1993). Low plasma GABA is a trait-like marker for bipolar illness. Neuropsychopharmacology.

[22] Li J., Zhang Z., Liu X., Wang Y., Mao F., Mao J., Lu X., Jiang D., Wan Y., Lv J.-Y. (2015). Study of GABA in healthy volunteers: Pharmacokinetics and pharmacodynamics. Front. Pharmacol..

[23] Wang S., Xiang Y.-Y., Zhu J., Yi F., Li J., Liu C., Lu W.-Y. (2017). Protective roles of hepatic GABA signaling in acute liver injury of rats. Am. J. Physiol. Gastrointest. Liver Physiol..

[24] Geisler C.E., Ghimire S., Bruggink S.M., Miller K.E., Weninger S.N., Kronenfeld J.M., Yoshino J., Klein S., Duca F.A., Renquist B.J. (2021). A critical role of hepatic GABA in the metabolic dysfunction and hyperphagia of obesity. Cell Rep..

[25] Geisler C.E., Ghimire S., Hepler C., Miller K.E., Bruggink S.M., Kentch K.P., Higgins M.R., Banek C.T., Yoshino J., Klein S. (2021). Hepatocyte membrane potential regulates serum insulin and insulin sensitivity by altering hepatic GABA release. Cell Rep..

[26] Olsen R.W., Sieghart W. (2008). International Union of Pharmacology. LXX. Subtypes of γ-aminobutyric acid(A) receptors: Classification on the basis of subunit composition, pharmacology, and function. Update. Pharmacol. Rev..

[27] Zhu S., Noviello C.M., Teng J., Walsh R.M., Kim J.J., Hibbs R.E. (2018). Structure of a human synaptic GABAA receptor. Nature.

[28] Sente A., Desai R., Naydenova K., Malinauskas T., Jounaidi Y., Miehling J., Zhou X., Masiulis S., Hardwick S.W., Chirgadze D.Y. (2022). Differential assembly diversifies GABAA receptor structures and signalling. Nature.

[29] Ben-Ari Y. (2002). Excitatory actions of GABA during development: The nature of the nurture. Nat. Rev. Neurosci..

[30] Bowery N.G., Bettler B., Froestl W., Gallagher J.P., Marshall F., Raiteri M., Bonner T.I., Enna S.J. (2002). International Union of Pharmacology. XXXIII. Mammalian γ-aminobutyric acid(B) receptors: Structure and function. Pharmacol. Rev..

[31] Tian J., Lu Y., Zhang H., Chau C.H., Dang H.N., Kaufman D.L. (2004). γ-Aminobutyric acid inhibits T cell autoimmunity and the development of inflammatory responses in a mouse type 1 diabetes model. J. Immunol..

[32] Prud’homme G.J., Glinka Y., Wang Q. (2015). Immunological GABAergic interactions and therapeutic applications in autoimmune diseases. Autoimmun. Rev..

[33] Dong H., Kumar M., Zhang Y., Gyulkhandanyan A., Xiang Y.-Y., Ye B., Perrella J., Hyder A., Zhang N., Wheeler M. (2006). Gamma-aminobutyric acid up- and downregulates insulin secretion from beta cells in concert with changes in glucose concentration. Diabetologia.

[34] Feng A.L., Xiang Y.Y., Gui L., Kaltsidis G., Feng Q., Lu W.Y. (2017). Paracrine GABA and insulin regulate pancreatic alpha cell proliferation in a mouse model of type 1 diabetes. Diabetologia.

[35] Menegaz D., Hagan D.W., Almaça J., Cianciaruso C., Rodriguez-Diaz R., Molina J., Dolan R.M., Becker M.W., Schwalie P.C., Nano R. (2019). Mechanism and effects of pulsatile GABA secretion from cytosolic pools in the human beta cell. Nat. Metab..

[36] Bhat R., Axtell R., Mitra A., Miranda M., Lock C., Tsien R.W., Steinman L. (2010). Inhibitory role for GABA in autoimmune inflammation. Proc. Natl. Acad. Sci. USA.

[37] Januzi L., Poirier J.W., Maksoud M.J.E., Xiang Y.-Y., Veldhuizen R.A.W., Gill S.E., Cregan S.P., Zhang H., Dekaban G.A., Lu W.-Y. (2018). Autocrine GABA signaling distinctively regulates phenotypic activation of mouse pulmonary macrophages. Cell. Immunol..

[38] Zhang B., Vogelzang A., Miyajima M., Sugiura Y., Wu Y., Chamoto K., Nakano R., Hatae R., Menzies R.J., Sonomura K. (2021). B cell-derived GABA elicits IL-10+ macrophages to limit anti-tumour immunity. Nature.

[39] Kang S., Liu L., Wang T., Cannon M., Lin P., Fan T.W.-M., Scott D.A., Wu H.-J., Lane A.N., Wang R. (2022). GAB functions as a bioenergetic and signalling gatekeeper to control T cell inflammation. Nat. Metab..

[40] Bao Z., Chen X., Li Y., Jiang W., Pan D., Ma L., Wu Y., Chen Y., Chen C., Wang L. (2023). The hepatic GABAergic system promotes liver macrophage M2 polarization and mediates HBV replication in mice. Antivir. Res..

[41] Li Y., Xiang Y.-Y., Lu W.-Y., Liu C., Li J. (2012). A novel role of intestine epithelial GABAergic signaling in regulating intestinal fluid secretion. Am. J. Physiol. Gastrointest. Liver Physiol..

[42] Xia Y., Chen S., Zhao Y., Chen S., Huang R., Zhu G., Yin Y., Ren W., Deng J. (2019). GABA attenuates ETEC-induced intestinal epithelial cell apoptosis involving GABAAR signaling and the AMPK-autophagy pathway. Food Funct..

[43] Tannahill G.M., Curtis A.M., Adamik J., Palsson-McDermott E.M., McGettrick A.F., Goel G., Frezza C., Bernard N.J., Kelly B., Foley N.H. (2013). Succinate is an inflammatory signal that induces IL-1β through HIF-1α. Nature.

[44] Toma I., Kang J.J., Sipos A., Vargas S., Bansal E., Hanner F., Meer E.J., Peti-Peterdi J. (2008). Succinate receptor GPR91 provides a direct link between high glucose levels and renin release in murine and rabbit kidney. J. Clin. Investig..

[45] Liu M., Ma N., Li S., Kang Z., Wang M., Wang D., Zhao J., Jiao H., Zhou Y., Wang X. (2025). *Prevotella*-produced succinate alleviates hepatic steatosis by enhancing mitochondrial function in layer-type chickens. J. Nutr..

[46] Xie L., Chen H., Zhang L., Yang Y.-Y., Zhou Y., Ma Y., Liu C., Wang Y.-L., Zhu Q., Yan Y.-J. (2026). Suppressing MASH fibrotic progression by blocking succinate-GPR91 signaling in HSCs. Hepatology.

[47] Tian J., Chau C., Hales T.G., Kaufman D.L. (1999). GABAA receptors mediate inhibition of T cell responses. J. Neuroimmunol..

[48] Sparrow E.L., James S., Hussain K., Beers S.A., Cragg M.S., Bogdanov Y.D. (2021). Activation of GABAA receptors inhibits T cell proliferation. PLoS ONE.

[49] Gilani A.A., Dash R.P., Jivrajani M.N., Thakur S.K., Nivsarkar M. (2014). Evaluation of GABAergic transmission modulation as a novel functional target for management of multiple sclerosis: Exploring inhibitory effect of GABA on glutamate-mediated excitotoxicity. Adv. Pharmacol. Sci..

[50] Rorsman P., Berggren P.-O., Bokvist K., Ericson H., Möhler H., Ostenson C.-G., Smith P.A. (1989). Glucose-inhibition of glucagon secretion involves activation of GABAA-receptor chloride channels. Nature.

[51] Ma X., Sun Q., Sun X., Chen D., Wei C., Yu X., Liu C., Li Y., Li J. (2018). Activation of GABAA receptors in colon epithelium exacerbates acute colitis. Front. Immunol..

[52] Gold M.S., Loeza-Alcocer E. (2024). Experimental colitis-induced visceral hypersensitivity is attenuated by GABA treatment in mice. Am. J. Physiol. Gastrointest. Liver Physiol..

[53] Gameiro A., Reimann F., Habib A.M., O’Malley D., Williams L., Simpson A.K., Gribble F.M. (2005). The neurotransmitters glycine and GABA stimulate glucagon-like peptide-1 release from the GLUTag cell line. J. Physiol..

[54] Nakamura U., Nohmi T., Sagane R., Hai J., Ohbayashi K., Miyazaki M., Yamatsu A., Kim M., Iwasaki Y. (2022). Dietary gamma-aminobutyric acid (GABA) induces satiation by enhancing the postprandial activation of vagal afferent nerves. Nutrients.

[55] Tian J., Dang H.N., Yong J., Chui W.S., Dizon M.P.G., Yaw C.K.Y., Kaufman D.L. (2011). Oral treatment with γ-aminobutyric acid improves glucose tolerance and insulin sensitivity by inhibiting inflammation in high fat diet-fed mice. PLoS ONE.

[56] Li X., Chen L., Zhu X., Lu Z., Lu Y. (2020). Effect of γ-aminobutyric acid-rich yogurt on insulin sensitivity in a mouse model of type 2 diabetes mellitus. J. Dairy Sci..

[57] Hosseini Dastgerdi A., Sharifi M., Soltani N. (2021). GABA administration improves liver function and insulin resistance in offspring of type 2 diabetic rats. Sci. Rep..

[58] Jin H., Han H., Song G., Oh H.J., Lee B.-Y. (2024). Anti-obesity effects of GABA in C57BL/6J mice with high-fat diet-induced obesity and 3T3-L1 adipocytes. Int. J. Mol. Sci..

[59] Ma X., Yan H., Hong S., Yu S., Gong Y., Wu D., Li Y., Xiao H. (2023). Gamma-aminobutyric acid promotes beige adipocyte reconstruction by modulating the gut microbiota in obese mice. Nutrients.

[60] Lee H.-Y., Lee G.-H., Hoang T.-H., Kim Y.-M., Jang G.-H., Seok C.-H., Gwak Y.-G., Lim J., Kim J., Chae H.-J. (2022). GABA and fermented *Curcuma longa* L. extract enriched with GABA ameliorate obesity through Nox4-IRE1α sulfonation-RIDD-SIRT1 decay axis in high-fat diet-induced obese mice. Nutrients.

[61] Begazo-Jimenez R., Yu A., Gros R., Lu W.-Y. (2025). Long-term supplementation of GABA regulates growth, food intake, locomotion, and lipid metabolism by increasing ghrelin and growth hormone in adolescent mice. Nutrients.

[62] Shan Y., Zhao J., Zheng Y., Guo S., Schrodi S.J., He D. (2023). Understanding the function of the GABAergic system and its potential role in rheumatoid arthritis. Front. Immunol..

[63] Soltani N., Qiu H., Aleksic M., Glinka Y., Zhao F., Liu R., Li Y., Zhang N., Chakrabarti R., Ng T. (2011). GABA exerts protective and regenerative effects on islet beta cells and reverses diabetes. Proc. Natl. Acad. Sci. USA.

[64] Ben-Othman N., Vieira A., Courtney M., Record F., Gjernes E., Avolio F., Hadzic B., Druelle N., Napolitano T., Navarro-Sanz S. (2017). Long-term GABA administration induces alpha cell-mediated beta-like cell neogenesis. Cell.

[65] Ackermann A.M., Moss N.G., Kaestner K.H. (2018). GABA and artesunate do not induce pancreatic alpha-to-beta cell transdifferentiation in vivo. Cell Metab..

[66] Berthoud H.-R., Neuhuber W.L. (2019). Vagal mechanisms as neuromodulatory targets for the treatment of metabolic disease. Ann. N. Y. Acad. Sci..

[67] Iwasaki Y., Sendo M., Dezaki K., Hira T., Sato T., Nakata M., Goswami C., Aoki R., Arai T., Kumari P. (2018). GLP-1 release and vagal afferent activation mediate the beneficial metabolic and chronotherapeutic effects of D-allulose. Nat. Commun..

[68] Wang S., Sui S., Liu Z., Peng C., Liu J., Luo D., Fan X., Liu C., Lu W.-Y. (2018). Protective roles of hepatic gamma-aminobutyric acid signaling in acute ethanol exposure-induced liver injury. J. Appl. Toxicol..

[69] Nagao T., Braga J.D., Chen S., Thongngam M., Chartkul M., Yanaka N., Kumrungsee T. (2024). Synergistic effects of peripheral GABA and GABA-transaminase inhibitory drugs on food intake control and weight loss in high-fat diet-induced obese mice. Front. Pharmacol..

[70] de Bie T.H., Witkamp R.F., Balvers M.G.J., Jongsma M.A. (2023). Effects of γ-aminobutyric acid supplementation on glucose control in adults with prediabetes: A double-blind, randomized, placebo-controlled trial. Am. J. Clin. Nutr..

[71] Martin A., Mick G.J., Choat H.M., Lunsford A.A., Tse H.M., McGwin G.G., McCormick K.L. (2022). A randomized trial of oral gamma aminobutyric acid (GABA) or the combination of GABA with glutamic acid decarboxylase (GAD) on pancreatic islet endocrine function in children with newly diagnosed type 1 diabetes. Nat. Commun..

[72] Tian J., Yong J., Dang H., Kaufman D.L. (2011). Oral GABA treatment downregulates inflammatory responses in a mouse model of rheumatoid arthritis. Autoimmunity.

[73] Jin Z., Korol S.V. (2023). GABA signalling in human pancreatic islets. Front. Endocrinol..

[74] Casado-Bedmar M., Roy M., Viennois E. (2023). The effect of sex-specific differences on IL-10^−/−^ mouse colitis phenotype and microbiota. Int. J. Mol. Sci..

